# Efficient and Specific Analysis of Red Blood Cell Glycerophospholipid Fatty Acid Composition

**DOI:** 10.1371/journal.pone.0033874

**Published:** 2012-03-30

**Authors:** Sabrina Klem, Mario Klingler, Hans Demmelmair, Berthold Koletzko

**Affiliations:** Division of Metabolic and Nutritional Medicine, Dr. von Hauner Children's Hospital, University of Munich Medical Center, Munich, Germany; Governmental Technical Research Centre of Finland, Finland

## Abstract

**Background:**

Red blood cell (RBC) n-3 fatty acid status is related to various health outcomes. Accepted biological markers for the fatty acid status determination are RBC phospholipids, phosphatidylcholine, and phosphatidyletholamine. The analysis of these lipid fractions is demanding and time consuming and total phospholipid n-3 fatty acid levels might be affected by changes of sphingomyelin contents in the RBC membrane during n-3 supplementation.

**Aim:**

We developed a method for the specific analysis of RBC glycerophospholipids. The application of the new method in a DHA supplementation trial and the comparison to established markers will determine the relevance of RBC GPL as a valid fatty acid status marker in humans.

**Methods:**

Methyl esters of glycerophospholipid fatty acids are selectively generated by a two step procedure involving methanolic protein precipitation and base-catalysed methyl ester synthesis. RBC GPL solubilisation is facilitated by ultrasound treatment. Fatty acid status in RBC glycerophospholipids and other established markers were evaluated in thirteen subjects participating in a 30 days supplementation trial (510 mg DHA/d).

**Outcome:**

The intra-assay CV for GPL fatty acids ranged from 1.0 to 10.5% and the inter-assay CV from 1.3 to 10.9%. Docosahexaenoic acid supplementation significantly increased the docosahexaenoic acid contents in all analysed lipid fractions. High correlations were observed for most of the mono- and polyunsaturated fatty acids, and for the omega-3 index (r = 0.924) between RBC phospholipids and glycerophospholipids. The analysis of RBC glycerophospholipid fatty acids yields faster, easier and less costly results equivalent to the conventional analysis of RBC total phospholipids.

## Introduction

The supply of n-3 long chain polyunsaturated fatty acids (LC-PUFA) is related to cardiovascular function, heart disease, morbidity and mortality, and in the perinatal period to child development [1], [2]. Commonly used biological markers for the dietary n-3 fatty acid intake are fatty acid composition of plasma or serum phospholipids (PL), triacylglycerides (TAG), cholesterol esters (CE), phosphatidylcholine (PC) and of red blood cell (RBC) PL, PC and phosphatidylethanolamine (PE) [3], [4]. The analysis of individual lipid species requires lipid separation usually by thin layer chromatography or solid phase extraction and the acid-catalysed derivatisation of fatty acids. In recent years, novel methods were developed avoiding these steps to reduce processing time and costs, and to increase sample throughput [5]. The application of these methods usually requires whole blood, plasma/serum, or RBC total lipids for the evaluation of the fatty acid status. Good correlations exist between fatty acid contents of these biological matrices and the diet. This is similar to correlations found between dietary fat intake and individual lipid classes [4].

Recently, our group developed a new, sensitive and robust method for the selective determination of plasma glycerophospholipids (GPL) fatty acids, which is independent of the postprandial state of a subject [6]. The conventional lipid extraction and separation has been replaced by the methanolic precipitation of proteins with co-precipitation of TAG and CE. In combination with a base catalysed synthesis of methyl esters at room temperature, this ensures the specific transesterification of GPL fatty acids [6]. In the past, it has been shown that extraction procedures cannot easily be transferred from plasma to RBC [7]. Therefore, it is essential to test the applicability of the plasma GPL method on RBC before its use in clinical studies.

The aim of the present study is to optimise and validate a new method for the analysis of GPL in RBC membranes, which avoids liquid-liquid extractions steps and chromatographic lipid class isolation. The application of the new method in a DHA supplementation trial and the comparison to established markers such as RBC PL, PC and PE will determine the relevance of RBC GPL as a valid fatty acid status marker in humans.

## Materials and Methods

### Subjects

Thirteen healthy subjects (6 males, 7 females) from the Munich area with an average age of 25.8±2.7 years (mean ± SD) and a BMI of 21.9±1.6 kg/m^2^ participated in an open-label, single-group assignment supplementation study with DHA. Taking supplements containing n-3 LC-PUFA or drugs interfering with the lipid metabolism were exclusion criteria as well as fatty fish consumption of more than once per week. The study was registered at ClinicalTrials.gov (NC T01192269). The Ethical Committee of the University of Munich Medical Center approved the study (034-10) and participants signed informed consent forms before they entered the study.

### Study design and supplements

Participants were asked to take a 950 µl DHASCO®-S microalgae oil capsule (Martek Biosciences, Columbia, MD) during breakfast daily over a period of 30 days. The capsule contained 520 mg DHA, but no other n-3 fatty acids. Participants were asked to record the intake of the capsules including time and date of consumption. Blood samples collected before and after the intervention period were analysed. This study was part of a larger project evaluating the DHA incorporation into plasma, RBC and buccal cell GPL.

### Sample preparation

After an overnight fasting period antecubital venous blood was collected into 7.5 ml EDTA-containing monovettes (Sarstedt, Nümbrecht, Germany). Samples were directly placed on ice and processed within 2 hours after collection. Plasma and RBC were separated by centrifugation (1000×g, 10 min, 4°C). RBC were washed 3 times with saline solution (0.9% NaCl). For the determination of PC and PE fatty acids aliquots of 500 µl RBC were haemolysed with 250 µl distilled water and suspended in 8 ml isopropanol containing BHT (0.05%). For the analysis of PL and GPL 100 µl aliquots of RBC were haemolysed with 100 µl distilled water and suspended in 260 µl methanol containing BHT (0.2%). All samples were stored at −80°C until analysis.

### GPL fatty acid analysis

The method of Glaser et al. for the analysis of plasma GPL was adapted for the determination of RBC GPL fatty acids [6], [8]. Intra- and inter-assay analyses were performed to validate the method before study commencement. In total 1.3 ml methanol and 100 µl of internal standard solution (14.6 mg PC15:0 in 100 ml methanol; Sigma Aldrich, Taufkirchen, Germany) were added to 200 µl haemolysed RBC. After continuous shaking on a Vibrax shaker (IKA, Stauffen, Germany) at 1000 rpm for 5 min samples were treated for 5 min in an ultrasound water bath (40 kHz, 120 W). The RBC suspension was centrifuged at 3030×g for 10 min at 4°C to separate the methanolic supernatant from cell fragments and precipitated proteins. After the transfer of the supernatant into a 4 ml brown glass vial synthesis of fatty acid methyl ester (FAME) was initiated by adding 50 µl sodium methoxide solution (25 wt% in methanol; Sigma Aldrich). The reaction was performed at room temperature and stopped after 4 min by adding 150 µl 3 M methanolic HCl (Sigma Aldrich). FAME were extracted twice with 600 µl hexane and the extracts were combined. Solvents were evaporated under a nitrogen flow and FAME redissolved in 50 µl hexane containing BHT (0.2%). Extracts were stored at −20°C until gas chromatographic (GC) analysis.

### Analysis of RBC PL fatty acids

1.8 ml chloroform, 540 µl methanol and 100 µl internal standard solution (PC15:0 in methanol) were added to a thawed RBC sample to obtain a chloroform-methanol ratio of 2∶1 v/v for lipid extraction [9]. A sodium chloride solution (2%) was added to the solvent mixture to obtain phase separation after subsequent centrifugation for 10 min at 3030×g and 4°C. The organic phase containing the lipids was dried under reduced pressure. The dried extract was redissolved in 400 µl chloroform/methanol (1∶1 v/v), applied on a 20×20 cm silica gel plate (Merck, Darmstadt, Germany) and lipid classes were separated using heptane, diisopropyl ether and acetic acid (60∶40∶3) as mobile phase [10]. Individual lipid bands were visualised with 2,7 di-chlor-fluorescein. The PL band was scraped off and transferred into a brown glass vial. FAME were synthesised in a closed vial with 3 N methanolic HCl at 85°C for 45 minutes. Samples were neutralised with a mixture of sodium carbonate, sodium hydrogen carbonate and sodium sulphate (1∶2∶2, Merck, KGaA). FAME were extracted twice with 1 ml hexane and redissolved in 50 µl hexane containing BHT (0.2%). Samples were stored at −20°C until GC analysis.

### Analysis of RBC PC and PE

The analysis of PC and PE in RBC membranes was performed as previously described by Geppert et al. [11]. Briefly, after extracting total lipids twice with 7 ml isopropanol/chloroform (3∶2 v/v) and 3 ml chloroform, the solvents were evaporated under reduced pressure. The separation of individual lipid fractions was achieved by thin layer chromatography using chloroform/methanol/ ammonia solution(25%)/distilled water (73∶27∶2,2∶2,8 by vol) as mobile phase. Corresponding PC and PE bands were scraped of the plate and transferred into 4 ml brown glass vials. FAME for GC analysis were obtained as describe above.

### Gas chromatography

FAME were quantified by GC with flame ionisation detection (Agilent 5890 series II, Waldbronn, Germany). The applied settings have previously been published by Glaser et al [6]. Peak integration was performed with EZChrom Elite (Version 3.1.7, Agilent).

### Statistical analysis

Relative fatty acid contents (% wt/wt) were calculated based on 20 cis-fatty acids and presented as mean and standard deviation. Precision analyses were performed by analyzing 8 aliquots of one RBC sample at the same day (intra-assay) or 26 aliquots over a period of 2 months (inter-assay) and calculated as coefficient of variation (CV). Intra-laboratory method performance were tested by comparing intra-assays (n = 8) of different staff members. Statistics for evaluating the effect of storage was performed with ANOVA repeated measures. The effect of DHA supplementation on fatty acid contents of different lipid fractions was assessed using paired t-tests. Relative DHA changes from baseline between PC, PE, GPL and PL were assessed with one-way ANOVA and Bonferroni post-hoc test. Correlations between fatty acid contents of different RBC compartments were evaluated according to Pearson. P-values <0.05 were considered to be statistically significant. All statistical analyses were computed using IBM SPSS Statistics for Windows, Version 19.0.0.1.

## Results

Table 1 shows the intra- and inter-assay data of RBC samples donated by different volunteers. The CV of the intra-assay evaluation (n = 8) ranged from 1.0 to 10.5% for all fatty acids and was <5% in most fatty acids. The inter-assay reproducibility (n = 26) was comparable to that of the intra-assay for all fatty acids (CV 1.3–10.9%), which contributed more than 0.5% to total fatty acids. Moreover, the inter-observer variability was tested by three different laboratory members, which achieved constantly a CV <10% for the 20 analysed RBC GPL fatty acids.

**Table 1:** Intra- and inter-assay reproducibility of the GPL fatty acid analysis.

	Intra-assay (n = 8)			Inter-assay (n = 26)		
	Mean	SD	CV [%]	Mean	SD	CV [%]
***Saturated Fatty Acids***						
C14:0	0.35	0.01	3.7	0.32	0.04	12.6
C16:0	22.99	1.01	4.4	23.31	0.80	3.4
C17:0	0.34	0.02	4.6	0.32	0.04	11.3
C18:0	18.34	0.27	1.5	17.92	0.40	2.3
***Monounsaturated Fatty Acids***						
C16:1n-7	0.29	0.02	7.6	0.35	0.03	8.6
C18:1n-7	1.45	0.03	2.0	1.42	0.15	10.9
C18:1n-9	14.07	0.22	1.5	13.38	0.22	1.6
C20:1n-9	0.29	0.00	3.3	0.27	0.01	4.9
***n-9 Polyunsaturated Fatty Acids***						
C20:3n-9	0.11	0.00	4.2	0.11	0.01	9.5
***n-6 Polyunsaturated Fatty Acids***						
C18:2n-6	11.84	0.50	4.3	12.34	0.16	1.3
C18:3n-6	0.06	0.00	3.7	0.08	0.01	10.1
C20:2n-6	0.22	0.01	6.4	0.25	0.01	5.3
C20:3n-6	2.37	0.02	1.0	2.21	0.04	1.7
C20:4n-6	15.39	0.84	5.5	15.57	0.55	3.5
C22:4n-6	2.37	0.25	10.5	3.20	0.20	6.3
C22:5n-6	0.67	0.02	3.0	0.64	0.03	5.0
***n-3 Polyunsaturated Fatty Acids***						
C18:3n-3	0.15	0.01	4.6	0.10	0.01	8.7
C20:5n-3	0.75	0.04	5.5	0.69	0.05	6.6
C22:5n-3	2.35	0.22	9.3	2.31	0.14	6.2
C22:6n-3	5.68	0.17	2.9	5.16	0.26	5.0

Mean and SD are expressed as %wt/wt.

**Figure 1:**
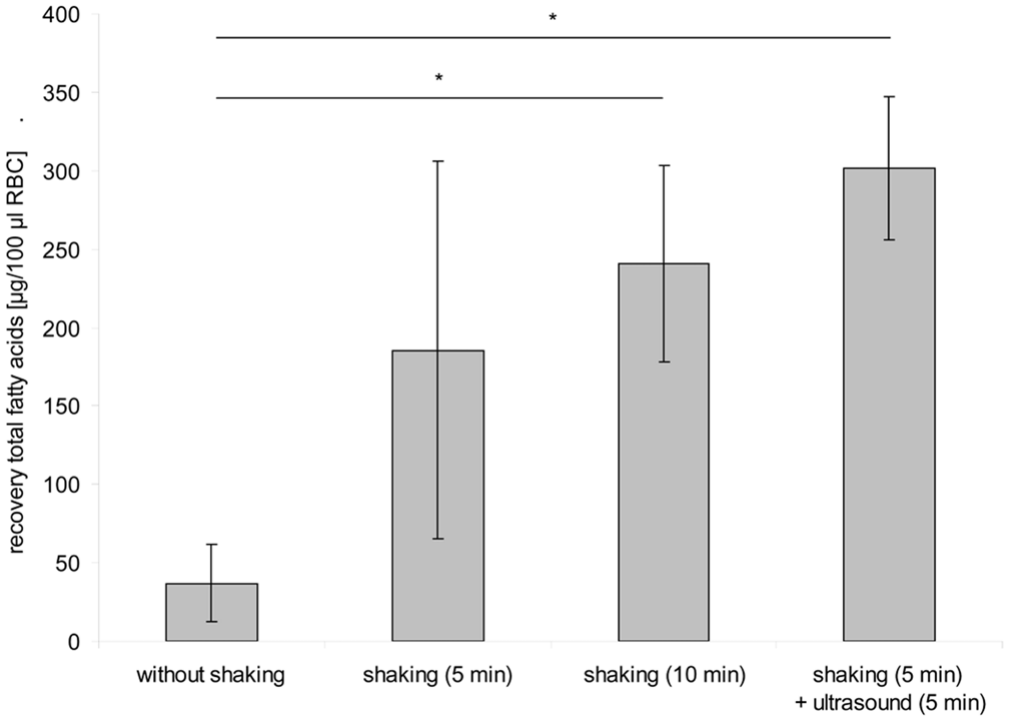
Recovery of total RBC fatty acids using different extraction procedures (*P*<0.05).

The extraction efficiency for of GPL was tested by applying 4 different extraction procedures (Figure 1). In total 16 aliquots (4×4) of an RBC sample were tested. Continuous shaking of RBC dissolved in methanol for 5 min yielded in 185±120 µg total GPL fatty acids per 100 µl RBC, which was similar to prolonged shaking for 10 min (241±63 µg) or additional ultrasound treatment for another 5 min (301±46 µg). Adding methanol to RBC without shaking caused clotting of RBC and lower recovery (37±24 µg). Partial clotting was also observed after shaking the samples for 5 or 10 min, but not when samples were treated with ultrasound. The fatty acid pattern of the 16 RBC aliquots did not differ to any appreciable extent (data not shown).

Contamination of the GPL containing supernatant with TAG and CE fatty acids was evaluated by separating lipid fractions in the methanolic supernatant via thin layer chromatography prior to the base-catalysed transesterification. Based on GPL total fatty acids the contamination originating from TAG fatty acids was 0.9% and from CE 0.4%. Only palmitic-, stearic-, oleic-, and linoleic acids derived from TAG or CE were detected.

Changes in fatty acid compositions caused by long-term storage are shown in Table 2. Washed RBC were kept in methanol containing BHT (0.2%) for 8 months at −80°C. Differences in fatty acid compositions were calculated based on changes relative to baseline and expressed in percent. Significant differences were found for some fatty acids, but values determined without storage were less than 10%, except for C17:0 (+29.5%), C18:3n-6 (+32.0%) and C22:4n-6 (+15.8%). The contribution of the first two fatty acids to total fatty acids was less than 0.5%. Palmitic acid was not affected under these storage conditions, whereas stearic acid decreased slightly (−2.8%). Fatty acids with a high potential for oxidative damage, such as n-3 and n-6 PUFA were affected differently. During storage the DHA percentage changed by 6.3%, (p = 0.061), whereas arachidonic acid (ARA) contents increased by 3.7% (p = 0.160), but differences were not statistically significant.

**Table 2:** Changes in fatty acids (%wt/wt) during storage of RBC samples (n = 13) in methanol over a period of 8 months at −80°C.

	Analysis without storage		Analysis after 8 months storage		Difference*	
	Mean	SD	Mean	SD	[%]	*P*
C14:0	0.28	0.07	0.29	0.08	4.09	*<0.001*
C16:0	22.62	1.21	22.81	1.00	0.85	*n.s.*
C17:0	0.33	0.04	0.42	0.08	29.50	*<0.001*
C18:0	17.75	0.55	17.26	0.58	−2.75	*<0.001*
C16:1n-7	0.35	0.13	0.37	0.14	6.90	*n.s.*
C18:1n-7	1.35	0.11	1.39	0.12	3.32	*n.s.*
C18:1n-9	15.04	0.89	14.73	0.74	−2.03	*0.040*
C20:1n-9	0.31	0.07	0.29	0.04	−7.59	*n.s.*
C20:3n-9	0.11	0.03	0.10	0.02	−3.45	*n.s.*
C18:2n-6	11.79	1.29	11.57	1.10	−1.90	*n.s.*
C18:3n-6	0.06	0.03	0.07	0.03	31.97	*0.014*
C20:2n-6	0.28	0.04	0.26	0.03	−6.41	*n.s.*
C20:3n-6	1.91	0.43	1.82	0.39	−4.42	*n.s.*
C20:4n-6	16.04	1.24	16.63	0.86	3.69	*n.s.*
C22:4n-6	2.76	0.44	3.19	0.52	15.79	*0.004*
C22:5n-6	0.78	0.15	0.70	0.15	−9.74	*0.048*
C18:3n-3	0.14	0.03	0.14	0.03	−1.15	*n.s.*
C20:5n-3	0.58	0.21	0.60	0.20	3.10	*n.s.*
C22:5n-3	2.04	0.31	2.22	0.35	8.44	*n.s.*
C22:6n-3	5.61	1.16	5.26	0.93	−6.30	*n.s.*

* Differences in fatty acid contents caused through sample storage were related to fatty acid contents of samples without storage. Mean and SD are expressed as %wt/wt. n.s.: not significant.

Table 3 shows the fatty acid composition of individual PL classes and total PL before and after the supplementation period. The micro algae oil supplementation increased DHA contents relative to baseline in PC by 92.3%±52.1, which was higher then in PE (33.2%±16.0), GPL (27.4%±16.5) and PL (13.3%±16.0) (ANOVA; *P*<0.001). ARA levels decreased during the supplementation period, which was significant in PL (−9.5%) and GPL (−3.9%), but not in PE (−0.6%) or PC (−6.9%). Similar results were shown for n-3 and n-6 docosapentaenoic acid (DPA). EPA was not affected by the supplementation, although by trend a slight increase could be observed in all fractions. At the beginning of the study contents of stearic acid in PC (10.7%±1.1) and PE (8.4%±1.1) were lower then in GPL (17.3%±0.5) or PL (18.5%±0.7), whereas oleic acid was more abundant in PC (17.2%±1.1) and PE (17.3%±0.9) then in GPL (14.6%±0.8) or PL (13.3%±0.8). All other GPL and PL fatty acid contents ranged between the levels of the respective fatty acids of PE and PC. The DHA supplementation had no effect on these observations.

**Table 3:** Effects of DHA supplementation on fatty acid composition (%wt/wt) of individual RBC PL fractions.

	PC					PE					GPL					PL				
	study start		study end			study start		study end			study start		study end			study start		study end		
	mean	SD	mean	SD	% diff	mean	SD	mean	SD	% diff	mean	SD	mean	SD	% diff	mean	SD	mean	SD	% diff
**C16:0**	36.2	1.3	36.0	1.4	−0.5	17.7	1.9	17.3	1.5	−2.6	22.4	1.1	22.8	0.8*	1.9	25.6	1.1	27.6	1.5*	8.0
**C18:0**	10.6	1.1	10.4	1.1	−1.6	8.4	1.1	8.1	0.5	−3.6	17.3	0.5	16.8	0.6**	−3.0	18.5	0.7	19.1	1.4	4.7
**C16:1n-7**	0.6	0.2	0.6	0.2	15.6	0.3	0.1	0.2	0.1	−6.7	0.4	0.1	0.4	0.1	−5.7	0.3	0.1	0.4	0.1	46.4
**C18:1n-7**	2.0	0.1	2.1	0.1	1.0	1.4	0.3	1.3	0.1	−9.0	1.4	0.1	1.3	0.1*	−5.3	1.2	0.2	1.1	0.1	−3.9
**C18:1n-9**	17.2	1.1	17.0	1.1	−1.4	17.3	0.9	17.0	1.2	−8.8	14.6	0.8	14.5	0.7	−0.7	13.3	0.8	13.1	0.7*	−1.7
**C20:3n-9**	0.1	0.0	0.1	0.0	−3.3	0.1	0.0	0.1	0.0	8.6	0.1	0.0	0.1	0.0	−5.8	0.1	0.0	0.1	0.0	31.1
**C18:2n-6**	19.5	1.6	19.7	1.6	1.2	5.6	0.9	5.4	0.9	−4.0	11.6	1.2	11.3	1.0	−2.1	10.2	1.0	9.9	0.8	−2.1
**C18:3n-6**	0.1	0.0	0.1	0.0	−6.4	0.1	0.0	0.1	0.0	0.4	0.1	0.0	0.1	0.0	−5.0	0.1	0.0	0.1	0.0	20.5
**C20:3n-6**	2.1	0.6	2.1	0.5	−2.9	1.3	0.3	1.2	0.2	−3.4	1.9	0.4	1.7	0.4***	−7.7	1.6	0.5	1.4	0.4***	−14.6
**C20:4n-6**	6.8	1.0	6.4	0.9	−5.8	25.4	1.9	25.5	1.4	−0.6	16.8	0.9	16.1	0.7*	−3.9	16.3	1.1	14.7	0.9***	−9.5
**C22:4n-6**	0.4	0.1	0.3	0.0	−6.9	8.2	1.1	7.8	1.0*	−6.0	3.1	0.6	3.2	0.5	2.2	3.7	0.4	3.1	0.4***	−16.2
**C22:5n-6**	0.2	0.1	0.1	0.0	−3.0	1.0	0.2	0.9	0.2**	−13.3	0.7	0.2	0.7	0.1***	−11.0	0.6	0.2	0.4	0.2***	−36.7
**C18:3n-3**	0.2	0.1	0.2	0.1	19.3	0.1	0.0	0.1	0.0	15.1	0.1	0.0	0.1	0.0	2.0	0.1	0.0	0.1	0.0	−9.7
**C20:5n-3**	0.5	0.4	0.5	0.1	27.5	1.0	0.3	1.0	0.3	4.3	0.6	0.3	0.6	0.1	9.3	0.6	0.2	0.6	0.2	9.5
**C22:5n-3**	0.5	0.1	0.4	0.1*	−12.5	4.5	0.6	4.2	0.6***	−7.9	2.3	0.4	2.1	0.3	−4.4	2.3	0.3	1.9	0.4**	−17.4
**C22:6n-3**	1.4	0.2	2.6	0.5***	92.3	6.2	0.9	8.3	0.9***	33.2	4.6	0.8	5.8	0.6***	27.3	4.3	0.8	4.9	0.9**	13.3

Differences between study start and end were based on baseline values. Paired t-test: *p<0.05, **p<0.01, *p<0.001.

Relationships of individual fatty acids between GPL and PC, PE or PL before and after the supplementation period are shown in Table 4. In general, SFA did not correlate between the different lipid fractions. At the beginning of the study high correlations were found for DHA between GPL/PE (r = 0.818) and GPL/PL (r = 0.940), and a good correlation between GPL/PC (r = 0.555). Similar correlations were shown for other n-3 and n-6 fatty acids, such as EPA, n-6 DPA and di-homo-γ-linolenic acid. GPL ARA contents were only correlated with PC ARA levels (r = 0.625), but not with ARA contents of the other fractions. The supplementation of micro algae oil influenced the correlations of individual fatty acids. The correlation of DHA between GPL/PE (r = 0.725) and GPL/PL (r = 0.729) was lower than at the beginning of the study. No correlations were found for DHA between GPL and PC. ARA, which did not correlate between GPL and PE showed a significant r-value of 0.657 after supplementation. The changes were most significant between GPL and PL as for most of the n-3 and n-6 fatty acid no longer correlations were found, except for DHA (r = 0.729), α-linolenic acid (r = 0.853) and di-homo-γ-linolenic acid (r = 0.874).

**Table 4:** Correlations between RBC GPL fatty acids and other RBC PL fractions before and after n-3 supplementation.

Correlation of individual GPL fatty acids *before study:*						
	*PC*		*PE*		*PL*	
	R	*P*	R	*P*	R	*P*
C16:0	0.525	n.s.	0.081	n.s.	0.343	n.s.
C18:0	0.529	n.s.	0.291	n.s.	0.162	n.s.
C16:1n-7	0.801	0.001	0.893	<0.001	0.874	<0.001
C18:1n-7	0.509	n.s.	0.725	0.005	0.409	n.s.
C18:1n-9	0.919	<0.001	0.441	n.s.	0.898	<0.001
C20:3n-9	0.352	n.s.	0.142	n.s.	0.743	0.006
C18:2n-6	0.797	0.001	0.446	n.s.	0.970	<0.001
C18:3n-6	0.436	n.s.	0.104	n.s.	0.427	n.s.
C20:3n-6	0.766	0.002	0.905	<0.001	0.967	<0.001
C20:4n-6	0.625	0.022	0.497	n.s.	0.473	n.s.
C22:5n-6	0.746	0.003	0.909	<0.001	0.820	0.001
C18:3n-3	0.930	<0.001	0.723	0.005	0.566	n.s.
C20:5n-3	0.899	<0.001	0.779	0.002	0.966	<0.001
C22:5n-3	0.739	0.004	0.704	0.007	0.594	0.042
C22:6n-3	0.555	0.049	0.818	<0.001	0.940	<0.001

Correlation of individual GPL fatty acids *after study:*						
	*PC*		*PE*		*PL*	
	R	*P*	R	*P*	R	*P*
C16:0	0.420	n.s.	0.281	n.s.	−0.073	n.s.
C18:0	0.529	n.s.	−0.363	n.s.	0.163	n.s.
C16:1n-7	0.943	<0.001	0.952	<0.001	0.313	n.s.
C18:1n-7	0.470	n.s.	0.547	n.s.	0.446	n.s.
C18:1n-9	0.878	<0.001	0.739	0.004	0.807	<0.001
C20:3n-9	0.341	n.s.	0.376	n.s.	−0.034	n.s.
C18:2n-6	0.524	n.s.	0.833	<0.001	0.498	n.s.
C18:3n-6	0.529	n.s.	0.489	n.s.	−0.141	n.s.
C20:3n-6	0.816	<0.001	0.913	<0.001	0.874	<0.001
C20:4n-6	0.681	0.010	0.657	0.015	0.427	n.s.
C22:5n-6	0.575	0.040	0.948	<0.001	0.226	n.s.
C18:3n-3	0.875	<0.001	0.761	0.003	0.853	<0.001
C20:5n-3	0.255	n.s.	0.946	<0.001	0.291	n.s.
C22:5n-3	0.577	0.039	0.885	<0.001	0.016	n.s.
C22:6n-3	0.454	n.s.	0.725	0.005	0.729	0.005

n.s. not significant.

**Figure 2:**
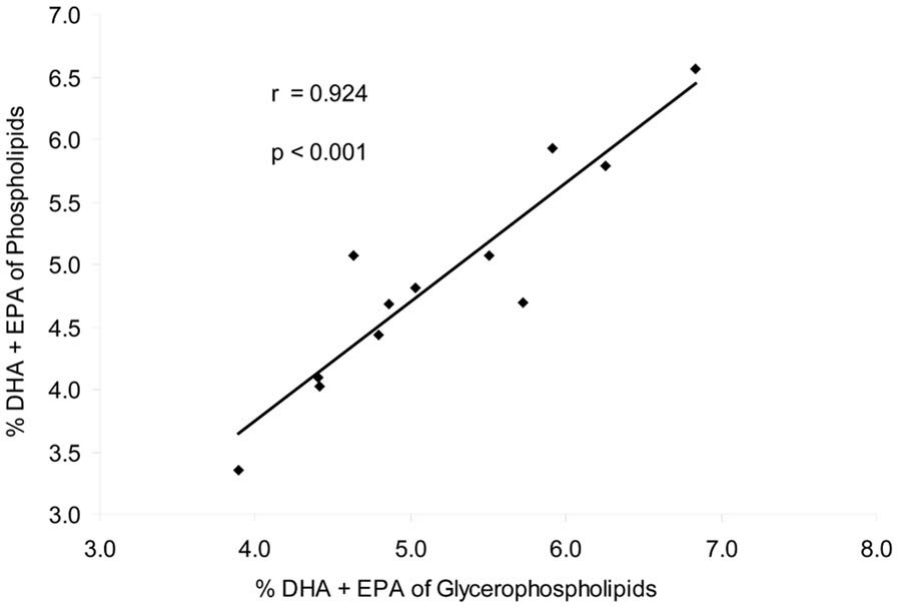
Correlation of RBC total PL and RBC GPL omega-3 index (n = 13).

Omega-3-indices based on GPL and PL were calculated from data determined at study start (Figure 2). The sum of EPA and DHA percentages was highly correlated between both lipid fractions (r = 0.924; *P*<0.001). At the end of the study a correlation of r = 0.780 (*P* = 0.002) was found.

## Discussion

This study shows that the analysis of RBC GPL is well suited for a fatty acid status determination in human. The base-catalyzed transesterification of RBC GPL fatty acids presented here has been applied for the analysis of plasma and cheek cell GPL before [6], [8], [12]. A good precision and robustness, a high sample throughput and a low-sample volume distinguish this method from fatty acid analyses using chromatographic separation of lipid fractions [13]. RBC GPL fatty acids before and after DHA supplementation were very similar to the fatty acid composition of total RBC PL analysed by the much more cumbersome conventional methodology.

A major challenge was clotting of RBC in methanol, which affected the total GPL fatty acid recovery. Extending the shaking time of RBC in methanol to 10 min had little effect on clot formation, but variations between measurements decreased compared to shorter shaking periods of 5 min. The application of ultrasound (indirect application in a water bath) after shaking resulted in a fine grained RBC suspension. The ultrasound treatment increased the recovery rate of the total fatty acid concentration and further decreased the variation of results between measurements. However, the PC standard did not compensate for the differences in extraction efficacy. We assume that the inclusion of PL into clots causes the loss of PL and not the partitioning of PL between solid and liquid phase during extraction, in line with the observation that fatty acid compositions were not affected. The ultrasound treatment is an integral part of the procedure to optimise recovery. Ultrasound treatment of 5 min seems to be sufficient to totally disperse the RBC clots in methanol.

For the analysis of GPL fatty acids in plasma an intra-assay CV of <3.7% and inter-assay CV of <10.7% was achieved for all studied fatty acids [6]. The precision of the fatty acid analysis in cheek cells was comparable, with CV ranging from 0.7% to 14.1% [12]. The precision data determined in this study for RBC were similar. This shows that the two step procedure, methanolic protein precipitation and base catalysed transesterification, is reliable for the GPL fatty acid determination in plasma, RBC, and cheek cells.

Storage of RBC samples over a longer period may be necessary in trials with large subject numbers [14]. Fatty acids of RBC samples are stable at temperatures below −50°C with or without free radical scavenging or iron binding agents [15]. Treating the washed RBC samples prior to freezing seems unnecessary, but when adding BHT a solvent is required as this antioxidant is insoluble in water. When our samples were stored in methanol containing BHT (0.05 mg/ml of RBC) for 8 months at −80°C, most of the fatty acid proportions did not change to an appreciable extent from pre-storage values. This is comparable with other published RBC conservation methods, which stored samples for 12 months or longer [14], [15], [16], [17]. However, a non significant trend towards a selective degradation of DHA was observed, and losses might become significant after 12 months of storage. Increasing the BHT concentration in the sample [15] or excluding the hemolysis of RBC with distilled water prior to freeze storage [14], [17] might improve the DHA stability, but this needs to be determined for GPL bound DHA.

The supplementation of micro algae oil, rich in DHA, significantly increased the DHA contents of PC and PE in the study subjects. The relative increase in PC was higher then in PE, which has also been described by other authors [11], [18], [19]. The non-uniform distribution of PC and PE in the RBC membrane and the different mechanisms for the fatty acid exchange of PC and PE with plasma may explain these observations.

SM in the RBC membrane behave differently during n-3 intervention and may affect the n-3 fatty acid status determination, as PC and PE proportions decrease and the SM proportion increases slightly with n-3 LC-PUFA supplementation [20]. This might be explained by the fact that the RBC membrane homeostasis is not only maintained by the exchange of other highly unsaturated fatty acids with DHA, i.e. ARA, but also by an increase of SM, which counteracts effects of high n-3 levels in PC or PE on membrane fluidity [21]. Our findings of increased SFA and decreased ARA contents in PL at the end of the study agree with this hypothesis.

Our data show a trend towards increased EPA levels after the supplementation of DHA in all studied fractions. This might be related to the retroconversion of DHA to EPA. In humans a retro conversation rate of at least 5% is observed [22]. This needs to be considered when DHA is given as the only n-3 fatty acid source.

The omega-3 index, based on the relative EPA+DHA content, is described as risk factor for coronary heart diseases [23]. We found a high correlation between the omega-3 index in RBC PL and GPL. It has to be determined, whether the omega-3 index proposed by Harris and von Schacky is comparable to our results, as different methods are applied and calculations of EPA+DHA might be based on different definitions of total fatty acids [23]. However, our results show a strong correlation between PL and GPL based omega-3 indices, therefore DHA and EPA proportions of GPL analysed with our method may be applicable for an omega-3 index determination.

In conclusion, the different responses of individual PL fractions to an n-3 supplementation have to be considered for the interpretation of changes in n-3 fatty acid status. Absolute and relative changes in RBC PC seem to be higher then in PE or GPL. The PL fatty acid composition is affected by different regulatory mechanisms in the RBC membrane, which affect the contribution of SM to total PL. Thus, we propose that our method for the analysis of RBC GPL fatty acids is advantageous over the RBC PL analysis, as changes of SM are excluded. Moreover, if the focus is not on a specific GPL subfraction, our method is an excellent alternative to monitor n-3 supplementation on fatty acid composition as it avoids the labour intensive, time-consuming and expensive separation of individual PL fractions.
